# Epidemiology and trends of hip fracture in centenarians: changes in clinical profile and in-hospital outcomes from a nationwide register study in Spain across 2004–2020

**DOI:** 10.1007/s40520-025-02994-w

**Published:** 2025-03-13

**Authors:** Juan Carlos Piñeiro-Fernández, Ramón Rabuñal-Rey, Eva Romay-Lema, Cristina Pedrosa-Fraga, David Rubal-Bran, Roi Suárez-Gil, Álvaro Marchán-López, Sonia Pértega-Díaz

**Affiliations:** 1 Internal Medicine Department, Lucus Augusti University Hospital, SERGAS. 1 Ulises Romero Street, Lugo, 27003 Spain; 2 Infectious Diseases Unit, Lucus Augusti University Hospital, SERGAS. 1 Ulises Romero Street, Lugo, 27003 Spain; 3 Department of Health Sciences, Faculty of Nursing and Podiatry, Universidade da Coruña. Rheumatology and Health Research Group, Esteiro, Ferrol, 15403 Spain; 4 Instituto de Investigación Biomédica de A Coruña (INIBIC), Nursing and Health Care Research Group, Xubias de Arriba 84, A Coruña, 15006 Spain

**Keywords:** Centenarians, Hip fractures, Comorbidity, Mortality, Epidemiologic studies, Electronic health records

## Abstract

**Background:**

Proximal hip fractures (PHFs) increased worldwide due to population ageing and represent the third cause of admission in Spanish centenarians. Recognizing trends in their evolution could improve their healthcare.

**Aim:**

To describe changes in trends in clinical characteristics, surgical decisions and in-hospital outcomes in PHF among centenarians in Spain, 2004 and 2020.

**Methods:**

This retrospective observational study included centenarians hospitalized with a principal diagnosis of PHF using data from the Hospital Discharge Records-Minimum Basic Data Set of the Spanish National Health System. Trends were analyzed using joinpoint regression analysis and descriptive and univariate statistics.

**Results:**

4,261 PHF admissions among centenarians were recorded. The number of PHF admissions increased from 147 in 2004 to 339 in 2020 (Average Percentage Change (APC)= 3.8%), with a higher increase in women. However, there was a reduction in the incidence of admissions in the last five years. Despite a significant increase in multimorbidity (from 44.4 to 64.1%) and in-hospital complications, there was a decreased in surgical delay (with more surgeries performed within 48 h: from 27.6 to 43.3%) and length of hospital stay (from 12.2 ± 8.6 to 9.7 ± 8.0 days), with a notable shift towards arthroplasty (from 28.7 to 52.7%), and stable mortality rates (APC=-1.5).

**Conclusion:**

This study indicates an increased complexity in patient profiles, with higher rates of multimorbidity and complications, but improvements in surgical care have led to reduced surgical delays and shorter hospital stays. Future studies are necessary to understand the factors associated with these trends and to design specific strategies in this vulnerable population.

**Supplementary Information:**

The online version contains supplementary material available at 10.1007/s40520-025-02994-w.

## Introduction

Proximal hip fracture (PHF) represents one of the most common and severe complications of osteoporosis and is an important public health problem [1]. Its incidence has increased worldwide due to population aging [2] and its prevalence among people aged 100 years or more is not surprising. This population segment has increased 89% in Spain in the last 20 years and PHF is the third leading cause of hospital admission in this age group [3]. 

This type of fracture has a markedly negative impact on quality of life and life expectancy [4], effects which are more pronounced in older individuals and in those with a high rate of comorbidities [5]. The creation of multidisciplinary units with co-management programs for patients with PHF has revolutionized the treatment of these patients [6], allowing for a greater number of interventions and a reduction in the average length of hospital stay, readmissions, complications, and mortality [7]. 

In a group as vulnerable as centenarians, it is common to have to make complex decisions that are challenging and involve important bioethical dilemmas when deciding whether or not to perform surgery [8]. Higher rates of complications (up to 50%, mostly infectious) and in-hospital mortality (up to 16%) have been reported in centenarian patients with PHF compared to slightly younger individuals [9, 10]. Despite this, recent studies have demonstrated the efficacy and safety of surgical treatment in these patients [11]. 

To date, there have been few studies of PHF in centenarians and most are single-center, have limited sample sizes, and report very heterogeneous results [12, 13]. This makes it complicated to evaluate their health outcomes [9]. Recognizing trends in the evolution and incidence of the main outcomes related to hospitalization due to PHF in centenarian patients (impact of comorbidities, type of surgery, surgical delay, and in-hospital complications) could help improve healthcare [14]. 

The aim of this study is to describe trends in the number of admissions for PHF among centenarians in Spain, changes in the clinical characteristics of these patients, and changes in the type of surgery and complications during hospitalization.

## Methods

### Study population and data source

This work uses similar methods to those used in an article previously published by this research group [3]. It is an observational, retrospective study of all patients aged 100 years or older hospitalized with a principal diagnosis of PHF in Spanish National Health System (SNS, for its initials in Spanish) hospitals from January 2004 to December 2020. The SNS provides universal health coverage, is decentralized in nature, and comprises the health services of Spain’s 17 autonomous communities and two autonomous cities, which have the capacity to manage their resources based on a common health framework [15]. 

The data for this study were obtained from the Hospital Discharge Records-Minimum Basic Data Set (HDR-MBDS). The HDR-MBDS is an anonymous, mandatory registry for all public and private hospitals that includes summarized, standardized information on all hospital discharges in a given year. It includes demographic data, primary clinical data, and information on variables related to the hospitalization itself.

Patients who were discharged with a principal diagnosis of PHF according to the International Classification of Diseases, Ninth Revision, Clinical Modification (ICD-9-CM) codes 820 (820.0-820.9) until 2016 and ICD-10-CM codes S72 (S72.0, S72.1, and S72.2) thereafter were identified. Patients were included only if they were hospitalized for an emergency admission. Those with complications from previous hip surgery, those who had fractures due to high-energy trauma, those who showed tumor etiology, and those with periprosthetic fractures were excluded.

Fractures were classified as intracapsular or extracapsular (pertrochanteric or subtrochanteric). Surgical procedures were classified as internal fixation (open reduction and internal fixation or closed reduction and internal fixation) or arthroplasty (hemiarthroplasty or total arthroplasty). The data on each admission described in this study included the year of hospitalization, sex, age, admitting department, procedure type (medical vs. surgical), secondary diagnoses (up to 20), procedures (up to 20), type of discharge (home, transfer to a residential facility, transfer to another hospital, or death), length of hospital stay (LOS), and date of surgery.

### Comorbidity analysis by reclassification of secondary diagnosis groups

The coding for secondary diagnoses and procedures was performed in the same manner as for the principal diagnosis using ICD-9-CM and ICD-10-CM codes. All secondary diagnoses were examined to analyze patient comorbidity, identify chronic diseases, and assess acute in-hospital complications. Supplementary Table 1 details the clustered specific conditions into 16 common disease groups classified according to the ICD-10-CM.

In line with the outcomes reported in several relevant investigations on multimorbidity [16], a total of 32 chronic diseases were reported in this study. Multimorbidity was defined as the presence of two or more chronic diseases in a single individual [16]. The Charlson Comorbidity Index (CCI) has been adapted for use with administrative databases and was used in this work to determine patient comorbidity. Severe comorbidity was defined as a score greater than 2 (which was associated with an in-hospital mortality rate greater than 50% per year) [17]. 

In-hospital complications included respiratory tract or urinary tract infections, acute respiratory failure, acute kidney injury, electrolyte disturbance, constipation or adynamic ileus, anemia, malnutrition, delirium, pressure ulcers, and pharmacologic or procedure-related complications.

To enhance the robustness of the statistical analysis and data visualization, database quality control techniques were performed. Variables that occurred with a frequency of less than 3% or deemed not clinically relevant in the study population were excluded from the final analysis. The complete list of excluded conditions is provided in Supplementary Table 1.

### Ethics

Access to the HDR-MBDS is universal and any researcher can request access from the Spanish Ministry of Health by filling out a form [15]. In accordance with Spanish legislation, all data requested from the HDR-MBDS are anonymized prior to receipt by the principal investigator. Therefore, it is not necessary to request individual patient consent or ethics committee approval.

### Statistical analysis

Trends in hospital admissions for PHF in the centenarian population from 2004 to 2020 were analyzed. The number of centenarian PHF admissions each year and the sex of each patient was obtained and shown both as the percentage of total and centenarian admissions and as the PHF admission rate among centenarians (calculated as the number of centenarian PHF admissions per 100 individuals aged ≥ 100 years).

To analyze trends, the total percentage increase in these figures was calculated by subtracting the 2004 data from the 2020 data, dividing the remainder by the 2004 data, and expressing the result as a percentage. In addition, a joinpoint log-linear regression analysis was performed to identify changes in trends and estimate the annual percentage change (APC) and 95% confidence intervals [18]. The analysis included up to three joinpoints and model fit and number of joinpoints were assessed using the Bayesian Information Criterion (BIC).

A descriptive analysis of the characteristics of patients admitted for PHF during the study period was also performed. Yearly detailed data are shown in the supplementary material. For the sake of brevity, three periods (2004–2010, 2011–2015, and 2016–2020) were defined for analyzing changes in patients’ baseline characteristics, surgical decisions, and in-hospital outcomes. Quantitative variables were reported as means, standard deviation, and medians whereas qualitative variables were shown as frequencies and percentages. A linear-by-linear association chi-square test was used to assess the correlation between year and categorical variables and Spearman’s correlation coefficient was used to analyze continuous data. Trends in the percentage of surgical patients, surgery type, delayed surgery, and the percentage of patients with a prolonged LOS and in-hospital mortality were also examined using joinpoint regression.

All analyses were two-tailed and values of *p* < 0.05 were considered significant. SPSS^®^ Version 28 software [19] and the Joinpoint Trend Analysis Software version 4.9.1.0 [20] were used for the statistical analysis.

## Results

### Trends in hip fracture admissions

**Fig. 1:**
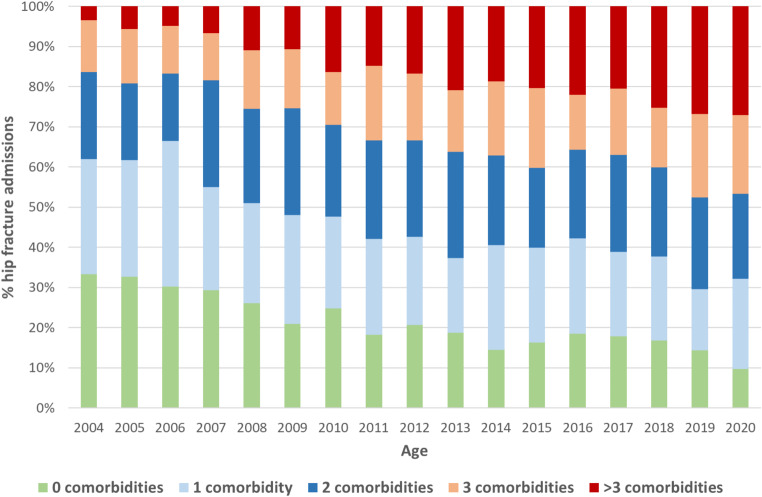
Trends in the hip fracture admissions in centenarians in Spain, by sex, 2004–2020

From 2004 to 2020, a total of 43,730 admissions of centenarians were recorded. Of them, 4,261 (9.7%) were due to proximal PHF, which represent 6.5 out of every 100,000 total hospital admissions and a PHF hospitalization rate of 2.1 out of every 100 centenarians/year. The number of centenarians admitted for PHF increased progressively from 147 hospitalizations in 2004 (4.2 × 100,000 total admissions) to 339 in 2020 (8.5 × 100,000 total admissions) (APC = 3.8%; 95% CI = 2.4%;5.3%). This increase was greater in women (from 6.4 to 16.9 × 100,000 total admissions, APC = 5.9%) than in men (from 1.7 to 3.5 × 100,000 total admissions, APC = 4.2%), with a female-to-male ratio among centenarians admitted with PHF of 5:1 in the total study period (Table 1. Figure 1. Supplementary Table 2).

**Table 1:** Trends in the hip fracture admissions in centenarians in Spain, by sex, 2004–2020. Joinpoint regression analysis

						TREND 1		TREND 2	
	2004	2010	2015	2020	% increase	Years	APC (95% CI)	Years	APC (95% CI)
No. hip fracture admissions	147	214	306	339	130.6%				
Males	28	46	47	57	103.6%				
Females	119	168	259	282	137.0%				
Ratio Female: Male	4.3	3.7	5.5	4.9	16.4%				
% Total admissions (x100,000)	4.2	5.8	8.2	8.5	103.1%	2004–2020	3.8 (2.4;5.3)*		
Males	1.7	2.6	2.6	3.5	105.6%	2004–2020	4.2 (2.9;5.4)*		
Females	6.4	8.5	13.3	16.9	164.8%	2004–2020	5.9 (4.7;7.1)*		
% Centenarians admissions (x100)	10.7%	9.7%	9.4%	9.8%	-8.3%	2004–2020	-1.5 (-2.4;-0.5)*		
Males	6.7%	7.5%	6.9%	7.2%	7.8%	2004–2020	0.2 (-0.9;1.3)		
Females	12.5%	10.6%	10.1%	10.6%	-15.1%	2004–2020	-2.1 (-3.3;-0.9)*		
% Hip admissions (x100 centenarians)	1.6%	2.3%	2.1%	2.0%	22.0%	2004–2008	11.5 (0.9–23.3)*	2008–2020	-1.7 (-3.2;-0.3)*
Males	1.1%	2.4%	1.6%	1.5%	40.2%	2004–2008	18.6 (2.8;36.8)*	2008–2020	-2.8 (-4.9;-0.6)*
Females	1.8%	2.3%	2.2%	2.1%	15.0%	2004–2020	-0.1 (-1.4;1.3)		

% increase: total percentage increase between 2004 and 2020; APC: Annual Percentage Change; CI: Confidence Interval (*p < 0.05)

However, there was a significant reduction in the proportion of PHF hospitalizations out of all centenarian admissions (APC=-1.5%; 95% CI=-2.4%;-0.5%) with no change in the incidence of PHF in female centenarians (APC=-0.1%; 95% CI=-1.4%;1.3%) and a downward trend in incidence in men starting in 2008 (APC=-2.8%; 95% CI=-4.9%;-0.6%) (Table 1. Figure 1. Supplementary Table 2).

### Changes in the baseline characteristics of centenarians with PHF

The characteristics of centenarians admitted for PHF each year during the study period as well as the surgical decisions and in-hospital outcomes are shown in Supplementary Table 3.

**Fig. 2:**
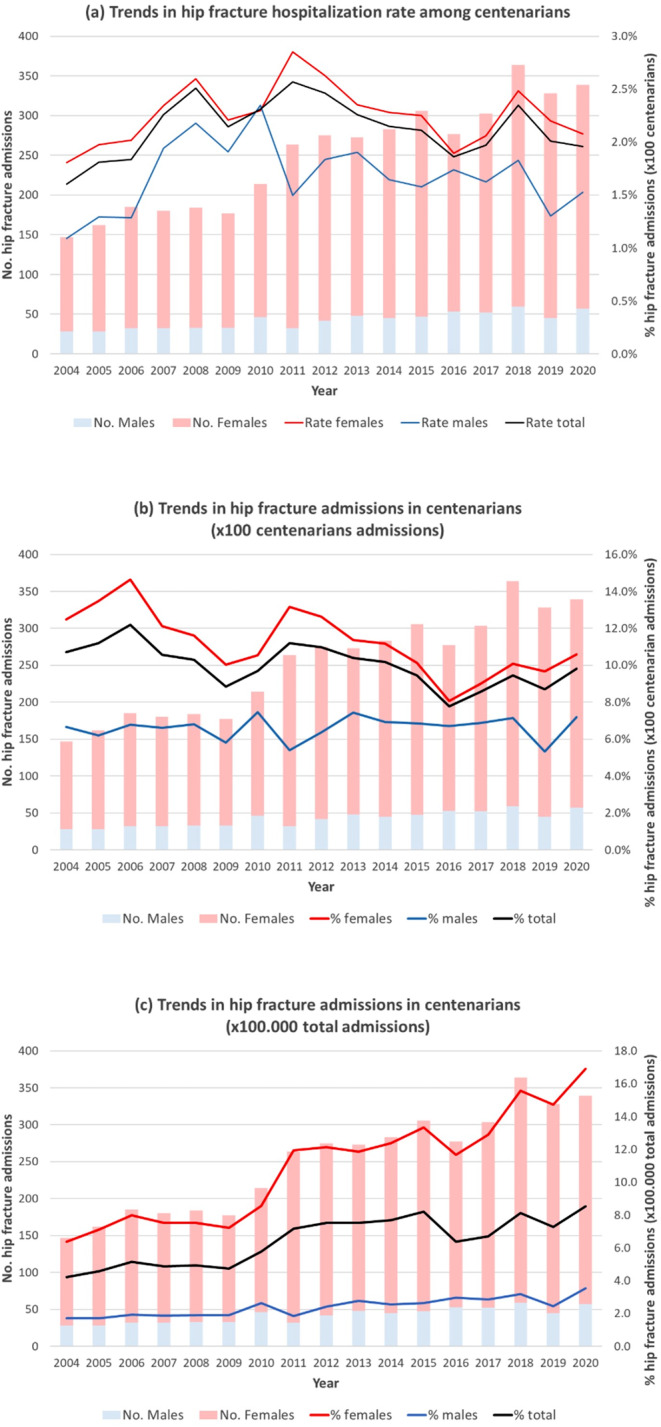
Trends in comorbidity among centenarians admitted with hip fracture in Spain, 2004–2020

During the study period, a trend toward greater comorbidity among centenarians admitted due to PHF was observed: 44.4% of patients had multimorbidity in 2004–2010 and 64.1% did in 2016–2020 (*p* < 0.001). Indeed, the proportion of patients with severe comorbidity increased from 6.2 to 15.1%, the average CCI score increased from 0.7 ± 1.0 to 1.1 ± 1.4, and the mean number of chronic diseases increased from 1.5 ± 1.4 to 2.4 ± 1.8 (Fig. 2).

The most prevalent comorbidities for the entire period were hypertension (42.5%), anemia (27.2%), dementia (13.8%), atrial fibrillation (12.3%), chronic kidney diseases (12.1%), and diabetes (9.8%) (Supplementary Table 1). The largest and most significant increases, in terms of relative percentage change, in baseline comorbidities were observed in cardiovascular-kidney-metabolic disease (dyslipemia, from 2.1% in 2004–2010 to 14.3% in 2016–2020; cerebrovascular disease, from 1.3 to 7.6%; chronic kidney disease, from 6.1 to 17.3%; atrial fibrillation, from 7.7 to 14.8%; diabetes, from 7.4 to 11%; and hypertension, from 35.8 to 44.3%), geriatric syndromes (dementia, from 11 to 16.4%; and vision loss, from 4.9 to 8.7%) and osteoporosis (from 4.6 to 8.8%) (Table 2).

**Table 2:** Changes in the characteristics of centenarians hospitalized for hip fracture during the study period

	2004–2010 (*n* = 1,249)	2011–2015 (*n* = 1,401)	2016–2020 (*n* = 1,611)	*p*
Age (years)	101.3 (1.6)	101.3 (1.5)	101.4 (1.7)	0.293
Sex, female	1017 (81.4)	1187 (84.7)	1345 (83.5)	0.182
Injury characteristics				0.421
Intracapsular fracture	504 (40.4)	576 (41.1)	681 (42.3)	
Pertrochanteric fracture	656 (52.5)	747 (53.3)	825 (51.2)	
Subtrochanteric fracture	89 (7.1)	78 (5.6)	105 (6.5)	
Department of admission				0.227
Traumatology	1197 (95.6)	1337 (95.4)	1512 (93.9)	
Geriatrics	29(2.3)	40 (2.9)	52 (3.2)	
Internal medicine	23 (1.8)	24 (1.7)	47 (2.9)	
Number of chronic diseases	1.5 ± 1.4 (1)	2.1 ± 1.6 (2)	2.4 ± 1.8 (2)	< 0.001
Multimorbidity	555 (44.4)	834 (59.5)	1033 (64.1)	< 0.001
Charlson Comorbidity index (CCI)	0.7 ± 1.0 (0)	0.9 ± 1.2 (0)	1.1 ± 1.4 (1)	< 0.001
Severe comorbidity (CCI ≥ 3)	77 (6.2)	164 (11.7)	243 (15.1)	< 0.001
Comorbidities				
Hypertension	447 (35.8)	653 (46.6)	713 (44.3)	< 0.001
Dyslipemia	26 (2.1)	96 (6.9)	231 (14.3)	< 0.001
Diabetes	93 (7.4)	146 (10.4)	177 (11.0)	0.002
Coronary heart disease	85 (6.8)	98 (7.0)	104 (6.5)	0.686
Heart failure	135 (10.8)	173 (12.3)	211 (13.1)	0.067
Atrial fibrillation	96 (7.7)	192 (13.7)	238 (14.8)	< 0.001
Other arrhythmias	49 (3.9)	54 (3.9)	63 (3.9)	0.992
Chronic pulmonary disease	87 (7.0)	76 (5.4)	110 (6.8)	0.988
Cerebrovascular disease	16 (1.3)	97 (6.9)	122 (7.6)	< 0.001
Chronic kidney disease	76 (6.1)	162 (11.6)	279 (17.3)	< 0.001
Thyroid disease	23 (1.8)	59 (4.2)	93 (5.8)	< 0.001
Dementia	138 (11.0)	185 (13.2)	264 (16.4)	< 0.001
Osteoarthrosis	59 (4.7)	102 (7.3)	138 (8.6)	< 0.001
Osteoporosis	58 (4.6)	90 (6.4)	141 (8.8)	< 0.001
Urinary incontinence	23 (1.8)	61 (4.4)	124 (7.7)	< 0.001
Visual loss	61 (4.9)	133 (9.5)	140 (8.7)	< 0.001
Hearing loss	109 (8.7)	125 (8.9)	164 (10.2)	0.171
Type of procedure				0.209
No surgery				
Surgery	1072 (85.8)	1231 (87.9)	1414 (87.8)	0.140
Internal fixation	756 (71.3)	840 (69.2)	374 (47.3)	< 0.001
Open reduction	272 (36.0%)	163 (19.4)	185 (49.5)	
Closed reduction	484 (64.0)	677 (80.6)	189 (50.5)	0.028
Arthroplasty	304 (28.7)	374 (30.8)	416 (52.7)	
Hemiarthroplasty	282 (93.1)	338 (96.0)	340 (81.7)	
Total arthroplasty	21 (6.9)	14 (4.0)	76 (18.3)	< 0.001
Surgical delay (days)*	3.5 ± 2.9 (3.0)	2.9 ± 2.6 (2.0)	2.4 ± 2.4 (2.0)	< 0.001
In-hospital complications				
Respiratory tract infection	48 (3.8)	56 (4.0)	96 (6.0)	0.006
Urinary tract infection	44 (3.5)	68 (4.9)	108 (6.7)	< 0.001
Acute respiratory failure	66 (5.3)	80 (5.7)	111 (6.9)	0.167
Acute kidney injury	49 (3.9)	120 (8.6)	152 (9.4)	< 0.001
Electrolyte disturbance	36 (2.9)	89 (6.4)	103 (6.4)	< 0.001
Constipation or adynamic ileus	41 (3.3)	88 (6.3)	122 (7.6)	< 0.001
Anemia	248 (19.9)	415 (29.6)	494 (30.7)	< 0.001
Transfusion	268 (21.5)	407 (29.1)	350 (21.7)	0.823
Urinary catheterization	47 (3.8)	61 (4.4)	59 (3.7)	0.832
Malnutrition	36 (2.9)	108 (7.7)	207 (12.9)	< 0.001
Delirium	45 (3.6)	98 (7.0)	152 (9.4)	< 0.001
Pressure ulcers	43 (3.4)	69 (4.9)	73 (4.5)	0.188
Pharmacologic or procedure-related complications	114 (9.1)	126 (9.0)	160 (9.9)	0.439
In-hospital mortality	207 (16.6)	198 (14.1)	234 (14.5)	0.151
Place of discharge, nursing home	57 (5.5)	114 (9.7)	156 (11.5)	< 0.001
Hospital length of stay (days)	12.2 ± 8.6 (11)	10.2 ± 9.0 (9)	9.7 ± 8.0 (8)	< 0.001
Survivors	12.6 ± 8.6 (11)	10.5 ± 9.2 (9)	9.7 ± 7.7 (8)	< 0.001
Non-survivors	10.3 ± 8.6 (8)	8.9 ± 7.2 (7)	9.3 ± 9.7 (7)	0.058

*Note*: Continuous variables are expressed as mean ± standard deviation (median) and categorical variables as number (percentage). * Percentage from the total of surgery (in 71.2% of patients)

No variations were observed according to the type of PHF or admitting department. In the study period, admissions for PHF were most common for extracapsular fractures (58.7% of total PHF) and patients were most often admitted to the traumatology department (94.7% of total admissions).

### Trends in surgical decisions, timing of surgery, and in-hospital outcomes

A total of 3,717 (87.2%) centenarians underwent surgery. No significant changes were observed in the proportion of patients who had surgery over the entire study period (85.8% in 2004–2010 vs. 87.8% in 2016–2020) (APC = 0.2%; 95% CI=-0.2%;0.5%), but there were changes in surgical decisions. The most common PHF repair surgery changed from internal fixation (which accounted for 71.3% of all procedures in 2004–2010 to 47.3% in 2016–2020) to arthroplasty (from 28.7 to 52.7% over the same periods). Most of the arthroplasty procedures were hemiarthroplasty, though there was a significant increase in the number of total arthroplasties (from 6.9% of all arthroplasty procedures in 2004–2010 to 18.3% in 2016–2020; *p* < 0.001) (Tables 2 and 3).

**Table 3:** Trends in the hip fracture surgery in centenarians in Spain. By sex, 2004–2020

						Trend 1		Trend 2	
	2004	2010	2015	2020	% increase	Years	APC (95% CI)	Years	APC (95% CI)
% Surgery	89.1	90.7	90.8	87.6	-2%	2004–2020	0.2 (-0.2;0.5)		
Males	82.1	91.3	89.4	86.0	5%	2004–2020	0.1 (-0.7;1.0)		
Females	90.8	90.5	91.1	87.9	-3%	2004–2020	0.1 (-0.2;0.5)		
% Arthroplasty	19.3	29.2	29.9	47.2	145%	2004–2014	2.7 (-0.7;6.2)	2014–2020	9.6 (4.4;15.1)*
Males	28.6	37.0	47.8	54.8	92%	2004–2020	4.8 (2.8;6.8)*		
Females	17.1	27.1	26.7	45.7	167%	2004–2020	5.6 (3.8;7.4)*		
% Surgeries delay ≤ 48 h	21.6	21.8	25.6	30.9	43%	2004–2020	2.8 (1.7;3.9)*		
Males	12.5	17.6	22.5	29.0	132%	2004–2020	3.9 (1.9;5.9)*		
Females	23.9	22.7	26.1	31.3	31%	2004–2020	2.5 (1.4;3.6)*		
% Patients with LOS > 7 days	71.4	72.0	59.8	50.7	-29%	2004–2007	2.9 (-0.9;6.9)	2007–2020	-2.9 (-3.3;-2.6)*
Males	67.9	73.9	68.1	54.4	-20%	2004–2020	-1.9 (-3.3;-0.5)*		
Females	72.3	71.4	58.3	50.0	-31%	2004–2020	-2.4 (-3.1;-1.8)*		
% In-hospital mortality	19.7	14.9	9.1	16.2	-18%	2004–2020	-1.5 (-3.4;0.5)		
Males	25.0	15.2	8.5	21.0	-16%	2004–2020	-2.8 (-5.9;0.5)		
Females	18.5	14.9	9.3	15.2	-18%	2004–2020	-1.1 (-3.3;1.0)		

*Note*: % increase: total percentage increase between 2004 and 2020; APC: Annual Percentage Change; CI: Confidence Interval (*p < 0.05)

Changes in the characteristics of patients for whom different surgical decisions were made are shown in Supplementary Table 4.

In addition, three other relevant trends were observed. First, there was a significant decrease in surgical delay over the study period (Supplementary Fig. 1). It declined from 3.5 ± 2.9 days in 2004–2010 to 2.4 ± 2.4 days in 2016–2020 (*p* < 0.001). In particular, an increase in the percentage of surgical procedures performed within the first 48 h was observed (27.6% in 2004–2010 vs. 43.3% in 2016–2020) (APC = 2.8%; 95% CI = 1.7%;3.9%) (Tables 2 and 3). On the other hand, a significant increase in morbidity burden (both the number of chronic diseases as well as the proportion of multimorbidity and severe comorbidity) and some in-hospital complications (urinary tract infection, acute respiratory failure, acute kidney injury, anemia, malnutrition, and delirium) was observed in patients who had a surgical delay of more than 48 h (Supplementary Table 5). Second, there was a significant decrease in LOS, from 12.2 ± 8.6 days in 2004–2010 to 9.7 ± 8.0 days in 2016–2020 (*p* < 0.001). Third, there was a significant increase in the percentage of centenarian patients discharged to a nursing home, from 5.5% in 2004–2010 to 11.5% in 2016–2020 (*p* < 0.001) (Tables 2 and 3).

Finally, there was a significant (*p* < 0.001) upward trend in the incidence of in-hospital complications (Table 2). The most pronounced trends were nosocomial infections (respiratory and urinary tract infections), acute kidney injury, electrolyte imbalance, constipation or adynamic ileus, anemia, malnutrition, and delirium. A total of 639 (15%) centenarians died during hospitalization, but in contrast to in-hospital complications, the overall mortality rate did not change over the study period (APC=-1.5; 95%CI=-3.4;0.5) (Tables 2 and 3; Supplementary Table 5).

## Discussion

This work identifies key trends in aspects regarding PHF in hospitalized centenarian patients in Spain between 2004 and 2020. During this period, there was a significant increase in the number of total admissions due to PHF, a trend which was more pronounced in women. However, in the last five years of the study, a reduction in the proportion of these admissions with respect to total admissions among centenarians has been identified, indicating a stabilization in their incidence. In addition, an increase in the burden of comorbidity and the proportion of in-hospital complications has been found despite improvements observed in surgical care. These findings underscore the increasing complexity of these patients and the need to establish specific strategies to prevent complications.

The number of PHFs has increased worldwide due to population aging, primarily in women [21], and is expected to double by 2050 [2]. Nevertheless, in recent years, some regions have observed a stabilization or decrease in its incidence in older adults [22–24], a trend confirmed in this study. Several hypotheses have been described to explain it, including early recognition and treatment of osteoporosis, preventive measures for falls, healthier habits, and an active lifestyle [25, 26]. The last point is especially relevant among centenarians, who maintain their daily activity for a longer time and have better functional status [27]. This results in early functional recovery at discharge and a reduction in the risk of new fractures due to frailty [28]. 

This research also reflects a tendency toward an increase in the morbidity burden. On the one hand, there was a 15.7% increase in the proportion of patients with multimorbidity. This figure is higher than the 10% observed in patients aged 72 to 88 years in the same time period [14]. In addition, it was found that the most common diseases and those that progress the most are cardiovascular diseases and dementia, in a similar proportion to what has previously been described [9]. These findings are logical, given that multimorbidity is closely related to age, and thus the greater demographic increase in centenarians makes them the age group with the greatest increase in age-associated comorbidities [16, 29]. On the other hand, a progressive increase in mean CCI score and severe comorbidity measured using the CCI was observed, with figures consistent with those reported in previous research [10, 26]. 

These findings are relevant for planning these patients’ care. Multiple studies have shown that comorbidity, as measured by the CCI, entails a greater risk of developing complications than other factors such as surgical delay and prolonged LOS [30–32]. Moreover, the occurrence of these complications continues to be the main risk factor for mortality in patients with PHF [33, 34], especially when they occur early after fracture [35]. In this sense, different trends were observed in this study that suggest an optimization of care processes in PHF.

First, although the decision for surgical management of PHF in individuals aged 90 years or older has traditionally posed a dilemma [8], recent studies have shown that a conservative approach is associated with more severe adverse outcomes during hospitalization and follow-up, particularly in patients with multimorbidity. These studies conclude that PHF surgery has a protective effect against the onset of complications [11, 36]. This work shows that almost 90% of the patients underwent surgery consistently over time and shows a trend toward arthroplasty as the most frequent procedure, which is in line with what has previously been published [37]. This change toward arthroplasty can be explained by technical advances and experience with the procedure in PHFs in this group of patients [4], which can lead to a lower complication rate, greater medium-term survival, and better functional recovery of the hip compared to internal fixation [38, 39]. 

Second, it shows a reduction in surgical delay and a progressive increase in the proportion of patients operated on in the first 48 h, which is in line with similar studies [40–42]. Several studies have demonstrated a significant association between surgical delay, an increase in certain complications (especially delirium), and mortality [13, 42] in octogenarians and nonagenarians, but not yet in centenarians [12, 41]. This study observed that patients with a surgical delay of more than 48 h tend to have a progressive increase in the burden of morbidity and certain complications (such as delirium, AKI, malnutrition, and anemia). However, a link to mortality cannot be established, since this goes beyond the scope of this research, which focuses on analyzing trends and not on determining factors associated with in-hospital events.

Third, a significant decrease in LOS was observed, a finding consistent with global trends in PHF [24]. However, it is noteworthy that the figures shown in this study and in previous research on centenarians with PHF, namely LOS between 8 and 14 days [10, 12], continue to be higher than in younger patients and those admitted for acute medical conditions, which reflects the complexity of PHF admissions in this patient group [43]. 

Despite these advances, this study shows that parallel to the increase in comorbidity, there is an upward trend in in-hospital complications, though the total mortality rate remains stable. Previous studies of centenarians have identified nosocomial infections (especially respiratory infections, followed by urinary infections), transfusions, delirium, or anemia as the most frequent complications. This was also described in this work, but with a higher proportion of them [40, 44]. In regard to in-hospital mortality, a higher rate was observed than what has previously been reported in studies of centenarians with PHF, which show figures between 8 and 12%, but the rate was stable over time [9, 26, 28]. 

Several interrelated factors may explain the trends in adverse hospital outcomes, although it is difficult to draw comparisons with other studies in centenarians due to significant methodological, geographical, and temporal differences [9]. On the one hand, improvements in preventive care of patients with PHF have facilitated the early detection of complications and may partially explain the greater number reported in this study. These improvements are implemented through co-management models, which promote shared decision-making, early surgical planning, and preoperative optimization of the clinical situation in the most complex patients. These measures have been shown to reduce surgical delay and improve post-surgical care by detecting and treating in-hospital complications early and effectively [6, 7]. Likewise, they can reduce or stabilize mortality [14, 41], as has been observed in our patients. On the other hand, regardless of improvements in surgical techniques and medical care, the inherent frailty of individuals who are the oldest old along with those who have a high number of chronic diseases [45] greatly increase the risk of in-hospital complications and mortality [4, 31]. 

Finally, centenarians are characterized by a concentration of morbidity in the last years of life and the onset of the most severe diseases when death is near [27], such as PHF (with an incidence of mortality four times higher in centenarians than in younger individuals) [41]. Therefore, despite observing a progressive decrease in surgical delay and LOS, mortality figures have remained stable [28]. 

In addition to the above, this study shows an upward trend in the proportion of discharges to nursing homes. Two factors may explain this. First, there is the implementation of hospital discharge policies that seek to improve the functional status of patients with PHF [4, 7], since 50% will not recover their previous level of function and require centers with specific care and resources. Second, there are the sociodemographic changes among Spanish centenarians; this age group has gone from living in the community in rural environments to nursing homes in urban environments [3]. However, the percentage increase is lower than what is described in other studies, which have reported figures as high as 40–60% [40, 41]. 

Several strengths and limitations must be considered when interpreting this study. The main advantage is that it is based on a long-term population-based cohort that reliably represents patients admitted for PHF in the Spanish population. The large sample size confers high statistical power, and it is the largest series of centenarians with PHF described in the literature. In addition, the data were extracted from hospital discharge records contained in a primary source of clinical information that is administrative, official, standardized, and represents practically all Spanish hospitals, which provides high external validity.

However, it is important to recognize the inherent limitations to the use of administrative databases, which are not designed for research purposes. The data quality depends on the accuracy of the coding, there may be errors in the recording of the data, and the analysis is limited by a lack of some meaningful information. In addition, the study’s descriptive, retrospective design limits the analysis of the factors associated with the trends observed. Further research with prospective studies that include long-term follow-up and evaluation of prognostic factors would allow for a deeper understanding of this work’s results. Likewise, these studies would also contribute to developing effective interventions to reduce complications in this increasingly complex and numerous age group.

## Conclusion

This study describes important trends in PHF among Spanish centenarians from 2004 to 2020, highlighting changes in epidemiological, clinical, surgical, and hospitalization-related outcomes. A progressive increase in the total number of admissions due to PHF in centenarians was observed, with a stabilization in the incidence in the last five years of study. In addition, it was confirmed that this group of patients has a growing morbidity burden. There was also an increase in the proportion of arthroplasties and a reduction in surgical delay. Finally, there was an increase in the proportion of in-hospital complications, although there was a decrease in the average length of stay and stable mortality throughout the period. These findings suggest significant improvements in the care of these patients, who are increasingly complex and vulnerable. These results also stress the need for specific strategies for preventing complications and planning healthcare resources to care for this growing population. New prospective, multicenter studies are needed to provide a deeper understanding of the factors associated with these trends.

## Electronic supplementary material

Below is the link to the electronic supplementary material.

Supplementary Material 1

## Data Availability

No datasets were generated or analysed during the current study.
