# Supplementary material for: Epidemiology and trends of hip fracture in centenarians: changes in clinical profile and in-hospital outcomes from a nationwide register study in Spain across 2004–2020
Source: Aging Clin Exp Res. 2025 Mar 13;37(1):84. doi: 10.1007/s40520-025-02994-w (PMC11903550; doi:10.1007/s40520-025-02994-w)
Supplement: Supplementary file 1 — Supplementary Material 1 [file 40520_2025_2994_MOESM1_ESM.pdf]

SUPPLEMENTARY MATERIAL

Supplementary Table 1. Secondary diagnosis clustering in comorbidity groups according to the ICD-10-CM for data analysis.

Group of diseases	Specific condition	Total (%)
Certain infectious and parasitic diseases (A00-B99)	Other infectious diseases (enteric infectious diseases, mycoses, viral infectious diseases, tuberculosis)	1.2%
	Other bacterial diseases not specified	1.2%
	Sepsis	0.4%
Neoplasms (C00-D49)	Personal history of previous malignant neoplasm	5.8%
	Active malignant neoplasm (solid organ neoplasm without metastases, solid organ neoplasm with metastases, haematological neoplasm)	2.6%
Diseases of the blood and blood-forming organs and certain disorders involving the immune mechanism (D50-D89)	Anemia	27.2%
Endocrine, nutritional and metabolic diseases (E00-E89)	Diabetes (diabetes with target organ damage, diabetes without target organ damage)	9.8%
	Malnutrition	8.2%
	Dyslipemia	7.4%
	Electrolyte disturbance	5.4%
	Thyroid disease	4.1%
	Obesity	0.7%
Mental, Behavioral and Neurodevelopmental disorders (F01-F99)	Dementia	13.8%
	Delirium	6.9%
	Other mental disorders	4.6%
	Depression and anxiety	2.8%
Diseases of the nervous system (G00-G99)	Movement disorders	1.4%
	Epilepsy	0.5%
Diseases of the eye and adnexa (H00-H59)	Visual loss	7.2%
Diseases of the ear and mastoid process (H60-H95)	Hearing loss	9.3%
Diseases of the circulatory system (I00-I99)	Hypertension	42.5%
	Atrial fibrillation	12.3%
	Heart failure	12.2%
	Coronary heart disease	6.7%
	Cerebrovascular disease	5.5%
	Other arrhythmias	3.9%
	Cardiac devices	3%
	Chronic peripheral venous insufficiency	2.5%
	Valvular heart diseases	2.2%
	Venous thromboembolic disease	2%
	Atherosclerosis/peripheral arterial disease/non-coronary artery disease	1.7%

Diseases of the respiratory system (J00-J99)	Chronic pulmonary disease (asthma or COPD)	6.4%
	Acute respiratory failure	6%
	Respiratory tract infection	4.7%
	Chronic respiratory failure	0.9%
Diseases of the digestive system (K00-K95)	Intra-abdominal infection	0.7%
	Chronic liver disease	0.4%
	Diverticular disease	1.5%
	Peptic gastro-duodenal or ulcerative disease	0.2%
	Constipation or adynamic ileus	5.9%
	Pathology of the gallbladder and biliary and pancreatic ducts	1.5%
	Gastro-intestinal hemorrhage	1.2%
	Other digestive disease	1.2%
Diseases of the skin and subcutaneous tissue (L00-L99)	Infections of the skin and subcutaneous tissue	0.2%
	Pressure ulcers	4.3%
Diseases of the musculoskeletal system and connective tissue (M00-M99)	Osteoarthrosis	7%
	Osteoporosis	6.8%
	Connective tissue diseases	0%
Diseases of the genitourinary system (N00-N99)	Chronic kidney disease	12.1%
	Acute kidney injury	7.5%
	Urinary tract infection	5.2%
	Urinary incontinence	4.9%
	Benign prostatic hyperplasia	2.9%
	Gynecological diseases	1.8%
External causes of morbidity (V00-Y99)	Pharmacologic or procedure-related complications	9.4%
	Tobacco use	1%
	Alcohol abuse	0%
Factors influencing health status and contact with health services (Z00-Z99)	Incidental fall	29.6%
	Place of residence, nursing home	14.2%
	Personal history of previous join replacement	2.5%
	Social problems	2%
	Admission to palliative care	
	Antimicrobial resistance	0.4%

Note: COPD: chronic obstructive pulmonary disease.

Supplementary Table 2. Trends in hip fracture admissions in centenarians in Spain, by sex, 2004-2020. Joinpoint regression analysis.

																				TREND 1		TREND 2	
		2004	2005	2006	2007	2008	2009	2010	2011	2012	2013	2014	2015	2016	2017	2018	2019	2020	%increase	Years	APC (95% CI)	Years	APC (95% CI)
No. hip fracture admissions	Total	147	162	185	180	184	177	214	264	275	273	283	306	277	303	364	328	339	130.6%				
	Males	28	28	32	32	33	33	46	32	42	48	45	47	53	52	59	45	57	103.6%				
	Females	119	134	153	148	151	144	168	232	233	225	238	259	224	251	305	283	282	137.0%				
	Ratio Female:Male	4.3	4.8	4.8	4.6	4.6	4.4	3.7	7.3	5.5	4.7	5.3	5.5	4.2	4.8	5.2	6.3	4.9	16.4%				
% Total admissions (x100,000)	Total	4.2	4.6	5.2	4.9	4.9	4.7	5.8	7.2	7.5	7.5	7.7	8.2	6.4	6.7	8.1	7.3	8.5	103.1%	2004-2020	3.8 (2.4;5.3)*		
	Males	1.7	1.7	1.9	1.9	1.9	1.9	2.6	1.8	2.4	2.8	2.6	2.6	3.0	2.8	3.2	2.4	3.5	105.6%	2004-2020	4.2 (2.9;5.4)*		
	Females	6.4	7.1	8.0	7.5	7.5	7.2	8.5	11.9	12.1	11.9	12.4	13.3	11.7	12.9	15.6	14.7	16.9	164.8%	2004-2020	5.9 (4.7;7.1)*		
% centenarians admissions (x100)	Total	10.7%	11.2%	12.2%	10.6%	10.3%	8.8%	9.7%	11.2%	11.0%	10.4%	10.2%	9.4%	7.8%	8.6%	9.4%	8.7%	9.8%	-8.3%	2004-2020	-1.5 (-2.4;-0.5)*		
	Males	6.7%	6.2%	6.8%	6.6%	6.8%	5.8%	7.5%	5.4%	6.4%	7.4%	6.9%	6.9%	6.7%	6.9%	7.2%	5.3%	7.2%	7.8%	2004-2020	0.2 (-0.9;1.3)		
	Females	12.5%	13.5%	14.7%	12.1%	11.6%	10.0%	10.6%	13.2%	12.6%	11.4%	11.2%	10.1%	8.1%	9.0%	10.1%	9.7%	10.6%	-15.1%	2004-2020	-2.1 (-3.3;-0.9)*		
% hip admissions per 100 centenarians	Total	1.6%	1.8%	1.8%	2.3%	2.5%	2.1%	2.3%	2.6%	2.5%	2.3%	2.1%	2.1%	1.9%	2.0%	2.3%	2.0%	2.0%	22.0%	2004-2008	11.5 (0.9-23.3)*	2008-2020	-1.7 (-3.2;-0.3)*
	Males	1.1%	1.3%	1.3%	1.9%	2.2%	1.9%	2.4%	1.5%	1.8%	1.9%	1.6%	1.6%	1.7%	1.6%	1.8%	1.3%	1.5%	40.2%	2004-2008	18.6 (2.8;36.8)*	2008-2020	-2.8 (-4.9;-0.6)*
	Females	1.8%	2.0%	2.0%	2.3%	2.6%	2.2%	2.3%	2.9%	2.6%	2.4%	2.3%	2.2%	1.9%	2.1%	2.5%	2.2%	2.1%	15.0%	2004-2020	-0.1 (-1.4;1.3)		

Note: % increase: total percentage increase between 2004 and 2020; APC: Annual Percentage Change; CI: Confidence Interval (*p<0.05).

Supplementary Table 3. Trends in the baseline characteristics. surgical decision and in-hospital outcomes in hip fracture admissions among centenarians, Spain, 2004-2020.

	2004	2005	2006	2007	2008	2009	2010	2011	2012	2013	2014	2015	2016	2017	2018	2019	2020	p
Age (years)	101.4+-1.7 (101.0)	101.0+-101.2 (1.5)	101.3+-1.7 (101.0)	101.2+-1.6 (101.0)	101.3+-1.7 (101.0)	101.2+-1.6 (101.0)	101.2+-1.6 (101.0)	101.3+-1.5 (101.0)	101.3+-1.5 (101.0)	101.4+-1.6 (101.0)	101.4+-1.5 (101.0)	101.3+-1.5 (101.0)	101.5+-1.8 (101.0)	101.3+-1.7 (101.0)	101.3+-1.7 (101.0)	101.5+-1.8 (101.0)	101.2+-1.7 (101.0)	0.508
Sex. female	81.0%	82.7%	82.7%	82.2%	82.1%	81.4%	78.5%	87.9%	84.7%	82.4%	84.1%	84.6%	80.9%	82.8%	83.8%	86.3%	83.2%	0.238
Injury characteristics																		
Pertrochanteric fracture	57.1%	50.6%	56.8%	51.7%	52.7%	49.2%	50.5%	53.8%	58.2%	48.7%	52.3%	53.6%	49.1%	53.5%	51.9%	54.0%	47.5%	
Subtrochanteric fracture	6.8%	4.3%	7.6%	5.6%	11.4%	7.3%	6.5%	4.5%	4.7%	4.8%	8.1%	5.6%	7.2%	4.3%	6.6%	7.0%	7.4%	
Intracapsular fracture	36.1%	45.1%	35.7%	42.8%	35.9%	43.5%	43.0%	41.7%	37.1%	46.5%	39.6%	40.8%	43.7%	42.2%	41.5%	39.0%	45.1%	
Admission at traumatology department	92.6%	95.5%	97.2%	93.8%	95.5%	95.3%	95.8%	94.5%	94.5%	95.4%	96.7%	92.4%	94.1%	94.5%	94.2%	93.8%	94.7%	
Number of chronic diseases	1.3+-1.3 (1.0)	1.0+-1.3 (1.3)	1.3+-1.2 (1.0)	1.4+-1.3 (1.0)	1.6+-1.4 (1.0)	1.7+-1.4 (2.0)	1.8+-1.6 (2.0)	2.0+-1.5 (2.0)	2.0+-1.6 (2.0)	2.2+-1.7 (2.0)	2.2+-1.6 (2.0)	2.2+-1.8 (2.0)	2.1+-1.8 (2.0)	2.2+-1.7 (2.0)	2.4+-1.9 (2.0)	2.5+-1.8 (2.0)	2.6+-1.8 (2.0)	<0.001
0	33.3%	32.7%	30.3%	29.4%	26.1%	20.9%	24.8%	18.2%	20.7%	18.7%	14.5%	16.3%	18.4%	17.8%	16.8%	14.3%	9.7%	
1	28.6%	29.0%	36.2%	25.6%	25.0%	27.1%	22.9%	23.9%	21.8%	18.7%	26.1%	23.5%	23.8%	21.1%	20.9%	15.2%	22.4%	
2	21.8%	19.1%	16.8%	26.7%	23.4%	26.6%	22.9%	24.6%	24.0%	26.4%	22.3%	19.9%	22.0%	24.1%	22.3%	22.9%	21.2%	
3	12.9%	13.6%	11.9%	11.7%	14.7%	14.7%	13.1%	18.6%	16.7%	15.4%	18.4%	19.9%	13.7%	16.5%	14.8%	20.7%	19.5%	
>3	3.4%	5.6%	4.9%	6.7%	10.9%	10.7%	16.4%	14.8%	16.7%	20.9%	18.7%	20.3%	22.0%	20.5%	25.3%	26.8%	27.1%	
Multimorbidity																		
Charlson Comorbidity index (CCI)	0.6+-1.2 (0.0)	0.0+-0.7 (0.9)	0.5+-0.8 (0.0)	0.6+-0.8 (0.0)	0.7+-1.0 (0.0)	0.8+-1.1 (0.0)	0.8+-1.1 (0.0)	0.8+-1.0 (0.5)	0.9+-1.4 (0.0)	1.0+-1.2 (1.0)	0.9+-1.3 (0.0)	1.0+-1.3 (1.0)	1.0+-1.4 (0.0)	0.9+-1.2 (1.0)	1.2+-1.4 (1.0)	1.2+-1.5 (1.0)	1.2+-1.4 (1.0)	<0.001
Severe comorbidity (CCI ≥ 3)	7.5%	6.8%	3.2%	2.2%	7.1%	8.5%	7.9%	9.5%	10.9%	13.2%	11.7%	13.1%	11.9%	9.6%	19.2%	17.7%	15.6%	<0.001
Comorbidities																		
Hypertension	29.9%	31.5%	30.8%	35.0%	35.3%	43.5%	42.1%	48.1%	42.2%	45.1%	47.7%	49.7%	45.5%	46.9%	37.6%	44.8%	47.5%	<0.001

Dyslipemia	0.0%	1.9%	2.2%	3.9%	1.1%	1.7%	3.3%	6.1%	3.6%	6.6%	8.5%	9.2%	8.3%	10.6%	15.1%	19.2%	17.1%	<0.001
Diabetes	6.1%	6.2%	7.6%	8.9%	3.8%	8.5%	10.3%	9.1%	10.5%	11.7%	7.8%	12.7%	12.3%	10.6%	8.5%	11.6%	12.4%	<0.001
Coronary heart disease	5.4%	14.2%	4.3%	10.6%	16.3%	10.2%	13.6%	9.1%	11.3%	18.3%	13.4%	9.8%	8.3%	12.5%	14.8%	13.4%	15.3%	0.922
Heart failure	6.8%	4.9%	5.4%	7.2%	8.2%	7.9%	7.0%	6.8%	6.2%	7.3%	7.4%	7.2%	8.3%	5.6%	5.2%	5.8%	7.7%	0.006
Atrial fibrillation	4.1%	7.4%	7.6%	5.0%	13.0%	6.8%	8.9%	19.3%	10.5%	12.5%	13.8%	12.7%	14.1%	11.6%	15.9%	13.7%	18.0%	<0.001
Other arrhythmias	3.4%	1.9%	5.9%	4.4%	3.8%	4.0%	3.7%	3.8%	4.4%	3.3%	3.2%	4.6%	4.0%	2.6%	4.4%	6.1%	2.4%	0.936
Chronic pulmonary disease	5.4%	7.4%	6.5%	6.1%	7.1%	9.6%	6.5%	5.3%	5.1%	6.2%	4.2%	6.2%	6.5%	4.0%	7.7%	8.8%	6.8%	0.731
Cerebrovascular disease	0.7%	1.2%	0.5%	0.0%	1.6%	2.3%	2.3%	8.0%	5.5%	5.9%	7.1%	8.2%	6.9%	5.0%	9.6%	8.2%	7.7%	<0.001
Chronic kidney disease	6.8%	7.4%	1.6%	2.8%	7.1%	9.0%	7.9%	9.1%	10.2%	11.4%	13.4%	13.4%	13.7%	12.9%	19.0%	21.6%	18.3%	<0.001
Thyroid disease	1.4%	1.2%	0.5%	1.1%	2.7%	3.4%	2.3%	2.7%	4.0%	4.0%	3.2%	6.9%	4.3%	4.6%	3.8%	5.5%	10.3%	<0.001
Dementia	15.0%	12.3%	14.1%	5.6%	12.0%	12.4%	7.5%	11.4%	16.0%	13.9%	12.7%	12.1%	15.9%	14.2%	18.4%	16.2%	16.8%	<0.001
Osteoarthrosis	4.8%	3.7%	5.4%	4.4%	2.7%	5.1%	6.5%	5.3%	7.6%	5.9%	9.5%	7.8%	5.4%	8.3%	9.6%	10.7%	8.3%	<0.001
Osteoporosis	4.1%	4.9%	3.8%	2.2%	3.8%	7.3%	6.1%	5.3%	5.5%	7.3%	6.7%	7.2%	7.6%	8.9%	8.8%	11.0%	7.4%	<0.001
Urinary incontinence	0.7%	3.1%	1.1%	2.2%	0.5%	2.3%	2.8%	4.2%	4.7%	4.8%	3.2%	4.9%	5.8%	5.9%	8.5%	10.1%	7.7%	<0.00
Visual loss	3.4%	4.3%	3.2%	3.3%	6.5%	5.6%	7.0%	4.9%	7.6%	9.9%	10.6%	13.7%	9.4%	5.9%	8.8%	9.1%	10.0%	<0.001
Hearing loss	12.2%	4.3%	8.1%	8.9%	9.8%	8.5%	9.3%	8.0%	8.0%	9.2%	9.2%	10.1%	9.7%	7.6%	12.9%	9.5%	10.6%	0.106
Surgery	89.1%	75.9%	85.4%	81.7%	88.0%	88.7%	90.7%	87.1%	89.8%	82.8%	88.3%	90.8%	85.6%	89.8%	88.2%	87.5%	87.6%	0.023
Surgery. internal fixation*	78.3%	73.8%	74.2%	63.4%	73.9%	69.9%	67.7%	72.2%	71.1%	67.4%	68.5%	67.0%	58.5%	47.5%	46.4%	43.5%	38.6%	<0.001
Open reduction	41.6%	43.3%	42.6%	40.2%	31.9%	28.4%	27.7%	27.4%	19.2%	18.1%	17.1%	15.7%	40.6%	41.9%	53.6%	66.7%	53.7%	
Closed reduction	58.4%	56.7%	57.4%	59.8%	68.1%	71.6%	72.3%	72.6%	80.8%	81.9%	82.9%	84.3%	59.4%	58.1%	46.4%	33.3%	46.3%	0.870
Surgery. arthroplasty*	21.7%	26.2%	25.8%	36.6%	26.1%	30.1%	32.3%	27.8%	28.9%	32.6%	31.5%	33.0%	41.5%	52.5%	53.6%	56.5%	61.4%	<0.001
Hemiarthroplasty	92.9%	93.8%	95.0%	94.3%	90.5%	93.6%	91.8%	96.8%	95.4%	94.1%	100.0%	94.4%	67.6%	74.7%	86.6%	94.3%	84.9%	
Total arthroplasty	7.1%	6.3%	5.0%	5.7%	9.5%	6.4%	8.2%	3.2%	4.6%	5.9%	0.0%	5.6%	32.4%	25.3%	13.4%	5.7%	15.1%	<0.001

Surgical delay (days)*	3.4+-2.7 (3.0)	3.0+-3.4 (2.8)	3.9+-3.4 (3.5)	3.9+-3.0 (3.0)	3.2+-3.0 (2.0)	3.2+-2.8 (3.0)	3.4+-2.6 (3.0)	2.9+-3.0 (2.0)	3.1+-2.5 (3.0)	2.9+-2.6 (2.0)	2.8+-2.4 (2.0)	2.8+-2.5 (2.0)	3.0+-3.0 (2.0)	2.4+-2.0 (2.0)	2.1+-2.3 (1.0)	2.3+-2.5 (2.0)	2.3+-2.3 (2.0)	<0.001
<24 hours	11.9%	9.1%	11.9%	9.6%	12.7%	13.7%	11.6%	11.1%	8.9%	18.1%	14.5%	17.7%	14.8%	15.7%	23.9%	22.0%	15.9%	
24-48 hours	15.6%	19.3%	16.9%	9.6%	24.6%	17.1%	16.3%	24.0%	19.6%	14.8%	23.0%	16.7%	20.0%	23.9%	27.2%	21.7%	28.8%	
48 hours – 3 days	15.6%	18.2%	10.2%	18.4%	17.8%	17.1%	12.2%	22.8%	20.2%	20.8%	15.2%	17.2%	22.2%	20.9%	16.2%	23.5%	20.7%	
3-4 days	16.5%	15.9%	11.0%	14.0%	9.3%	15.4%	17.0%	11.7%	15.5%	13.4%	15.8%	19.3%	9.6%	15.3%	12.6%	13.4%	12.2%	
4-5 days	14.7%	11.4%	13.6%	14.9%	8.5%	11.1%	12.2%	12.9%	13.7%	10.7%	12.1%	10.9%	12.2%	11.2%	9.1%	7.6%	10.2%	
5-6 days	8.3%	5.7%	7.6%	11.4%	8.5%	7.7%	15.0%	6.4%	6.5%	7.4%	7.3%	6.8%	6.1%	4.9%	3.9%	4.0%	3.4%	
6-7 days	5.5%	9.1%	10.2%	6.1%	5.9%	6.8%	5.4%	2.3%	7.1%	4.7%	3.6%	2.6%	5.2%	2.2%	3.2%	3.2%	4.4%	
>=7 days	11.9%	11.4%	18.6%	15.8%	12.7%	11.1%	10.2%	8.8%	8.3%	10.1%	8.5%	8.9%	10.0%	6.0%	3.9%	4.7%	4.4%	
In-hospital complications																		
Respiratory tract infection	2.7%	4.9%	4.3%	3.3%	3.3%	6.2%	2.3%	3.8%	2.9%	4.8%	3.5%	4.9%	4.7%	6.3%	6.3%	6.7%	5.6%	0.006
Urinary tract infection	3.4%	4.3%	2.2%	2.2%	6.5%	3.4%	2.8%	4.9%	4.4%	5.5%	6.0%	3.6%	6.5%	7.3%	4.1%	7.3%	8.6%	<0.001
Acute respiratory failure	4.1%	5.6%	5.4%	4.4%	7.1%	5.1%	5.1%	4.5%	4.7%	8.4%	4.6%	6.2%	6.5%	7.3%	8.5%	7.3%	4.7%	0.084
Acute kidney injury	0.7%	3.1%	1.1%	2.8%	3.8%	6.2%	8.4%	5.7%	10.2%	10.3%	8.8%	7.8%	7.9%	5.6%	11.8%	11.3%	9.7%	<0.001
Electrolyte disturbance	2.0%	3.7%	0.0%	3.3%	3.8%	3.4%	3.7%	4.9%	6.5%	8.1%	5.7%	6.5%	6.1%	5.3%	7.4%	5.8%	7.1%	<0.001
Constipation or adynamic ileus	2.7%	3.1%	2.7%	2.8%	3.3%	2.3%	5.6%	4.5%	5.5%	7.0%	8.8%	5.6%	7.2%	7.9%	6.9%	9.1%	6.8%	<0.001
Anemia	14.3%	17.9%	16.2%	18.9%	23.9%	24.9%	21.5%	24.2%	28.0%	35.5%	30.7%	29.4%	28.2%	30.4%	30.2%	35.7%	28.6%	<0.001
Transfusion	19.0%	19.8%	17.3%	18.9%	26.1%	24.9%	23.4%	27.3%	28.0%	33.0%	30.4%	26.8%	14.8%	15.8%	22.5%	29.9%	23.9%	0.227
Urinary catheterization	1.4%	3.1%	4.9%	3.9%	4.9%	3.4%	4.2%	2.7%	5.5%	4.0%	5.3%	4.2%	4.7%	2.3%	3.6%	4.3%	3.5%	0.833
Malnutrition	0.7%	2.5%	0.0%	4.4%	3.3%	3.4%	5.1%	6.4%	5.8%	7.7%	6.4%	11.8%	9.7%	9.9%	14.3%	16.8%	12.7%	<0.001
Delirium	2.0%	1.2%	2.7%	3.9%	4.3%	4.0%	6.1%	3.0%	5.1%	7.0%	9.9%	9.5%	10.1%	8.3%	9.3%	11.6%	8.0%	<0.001
Pressure ulcers	0.0%	2.5%	4.3%	2.8%	5.4%	2.8%	5.1%	5.3%	5.1%	5.1%	6.7%	2.6%	4.0%	5.6%	2.7%	5.2%	5.3%	0.090
Pharmacologic or procedure-related complications	11.6%	8.0%	9.7%	6.7%	9.2%	9.0%	9.8%	6.8%	9.8%	8.8%	9.2%	10.1%	10.8%	11.2%	7.4%	9.8%	10.9%	0.415

In-hospital mortality	19.70%	17.30%	20.50%	13.90%	11.40%	19.20%	15.00%	14.80%	17.50%	17.20%	12.70%	9.20%	11.20%	15.80%	15.90%	12.80%	16.20%	0.051
Place of discharge. nursing home	0.9%	1.5%	4.1%	8.4%	8.0%	7.0%	6.6%	7.2%	8.4%	7.4%	10.4%	14.2%	10.7%	10.6%	16.2%	11.7%	7.6%	<0.001
Hospital stay (days)	11.3+-6.0 (11.0)	11.0+-12.6 (11.3)	12.1+-8.0 (11.0)	13.0+-9.2 (11.0)	13.4+-9.7 (11.0)	11.2+-6.6 (10.0)	11.7+-8.2 (10.0)	11.2+-14.7 (9.0)	10.5+-7.6 (9.0)	10.1+-7.0 (9.0)	9.8+-7.2 (8.0)	9.8+-6.4 (9.0)	9.9+-6.7 (8.0)	9.7+-6.0 (9.0)	9.6+-7.5 (8.0)	10.0+-11.0 (8.0)	9.3+-7.8 (8.0)	<0.001
Survivors	12.0+-6.0 (12.0)	12.0+-13.0 (11.0)	13.0+-8.0 (11.0)	13.0+-9.0 (12.0)	14.0+-10.0 (12.0)	11.0+-6.0 (10.0)	12.0+-9.0 (10.0)	12.0+-15.0 (9.0)	11.0+-7.0 (9.0)	10.0+-7.0 (9.0)	10.0+-7.0 (9.0)	10.0+-7.0 (9.0)	10.0+-7.0 (8.0)	10.0+-6.0 (9.0)	9.0+-6.0 (8.0)	10.0+-11.0 (8.0)	9.0+-8.0 (8.0)	<0.001
Non-survivors	10.0+-7.0 (8.0)	8.0+-11.0 (11.0)	11.0+-8.0 (9.0)	11.0+-13.0 (9.0)	11.0+-9.0 (8.0)	10.0+-7.0 (9.0)	9.0+-5.0 (9.0)	9.0+-9.0 (6.0)	10.0+-8.0 (8.0)	9.0+-7.0 (8.0)	8.0+-6.0 (7.0)	8.0+-5.0 (7.0)	9.0+-7.0 (7.0)	9.0+-8.0 (6.0)	10.0+-14.0 (7.0)	10.0+-11.0 (7.0)	8.0+-7.0 (7.0)	0.054

Note: Continuous variables are expressed as mean ± standard deviation (median) and categorical variables as number (percentage). * Percentage of total number of surgeries (71.2% of patients).

Supplementary Table 4. Trends in the basal characteristics, timing of surgery and in-hospital outcomes according to surgery decision. Hip fracture admissions in centenarians in Spain, 2004-2020.

	No surgery							Internal fixation							Arthroplasty						
	2004-2010		2011-2015		2016-2020		p	2004-2010		2011-2015		2016-2020		p	2004-2010		2011-2015		2016-2020		p
Age (years)	101.4±1.7	101	101.5±1.7	101	101.6±1.9	101	0.382	101.3±1.6	101	101.3±1.5	101	101.3±1.6	101	0.672	101.2±1.6	101	101.3±1.4	101	101.2±1.5	101	0.766
Sex. female	138	78.0%	138	81.2%	159	80.7%	0.518	638	84.4%	739	88.0%	323	86.4%	0.190	229	75.3%	295	78.9%	317	76.2%	0.866
Injury characteristics							0.547							0.070							0.536
Subtrochanteric fracture	5	2.8%	5	2.9%	8	4.1%		83	11.0%	71	8.5%	41	11.0%		1	0.3%	0	0.0%	0	0.0%	
Pertrochanteric fracture	68	38.4%	63	37.1%	64	32.5%		578	76.5%	668	79.5%	265	70.9%		6	2.0%	9	2.4%	14	3.4%	
Intracapsular fracture	104	58.8%	102	60.0%	125	63.5%		95	12.6%	101	12.0%	68	18.2%		297	97.7%	365	97.6%	402	96.6%	
Number of chronic diseases	1.6±1.3	1	2.4±1.7	2	2.3±1.7	2	<0.001	1.5±1.3	1	2.1±1.6	2	2.3±1.8	2	<0.001	1.5±1.5	1	2±1.7	2	2.3±1.7	2	<0.001
0	40	22.6%	25	14.7%	30	15.2%		214	28.3%	140	16.7%	67	17.9%		92	30.3%	80	21.4%	60	14.4%	
1	57	32.2%	34	20.0%	44	22.3%		208	27.5%	200	23.8%	75	20.1%		78	25.7%	85	22.7%	96	23.1%	
2	41	23.2%	37	21.8%	45	22.8%		174	23.0%	202	24.0%	80	21.4%		64	21.1%	81	21.7%	99	23.8%	
3	23	13.0%	31	18.2%	34	17.3%		103	13.6%	159	18.9%	65	17.4%		36	11.8%	59	15.8%	72	17.3%	
>3	16	9.0%	43	25.3%	44	22.3%		57	7.5%	139	16.5%	87	23.3%		34	11.2%	69	18.4%	89	21.4%	
Multimorbidity	80	45.2%	111	65.3%	123	62.4%	<0.001	334	44.2%	500	59.5%	232	62.0%	<0.001	134	44.1%	209	55.9%	260	62.5%	<0.001
Charlson Comorbidity index (CCI)	1±1.1	1	1.4±1.5	1	1.2±1.5	1	0.408	0.5±0.9	0	0.8±1.2	0	1.1±1.5	1	<0.001	0.7±1.1	0	0.8±1.2	0	1.1±1.4	1	<0.001

Severe comorbidity (CCI ≥ 3)	19	10.7%	39	22.9%	30	15.2%	0.276	37	4.9%	85	10.1%	52	13.9%	<0.001	20	6.6%	38	10.2%	71	17.1%	<0.001
Surgical delay (days)*	-	-	-	-	-	-	-	3.4±3	3	2.8±2.6	2	2.4±2.7	2	<0.001	3.5±2.6	3	3.2±2.6	3	2.6±2.3	2	<0.001
<24 horas	-	-	-	-	-	-	-	73	12.8%	96	16.8%	78	21.7%		19	8.4%	22	8.6%	54	13.3%	
[24-48) horas	-	-	-	-	-	-	-	98	17.1%	110	19.3%	96	26.7%		39	17.2%	54	21.0%	96	23.6%	
>=48 horas	-	-	-	-	-	-	-	401	70.1%	365	63.9%	185	51.5%		169	74.4%	181	70.4%	257	63.1%	
In-hospital complications																					
Respiratory tract infection	11	6.2%	6	3.5%	12	6.1%	0.988	33	4.4%	34	4.0%	17	4.5%	0.965	3	1.0%	16	4.3%	25	6.0%	<0.001
Urinary tract infection	9	5.1%	10	5.9%	14	7.1%	0.711	22	2.9%	37	4.4%	26	7.0%	0.002	12	3.9%	20	5.3%	26	6.3%	0.177
Acute respiratory failure	16	9.0%	18	10.6%	13	6.6%	0.384	35	4.6%	39	4.6%	19	5.1%	0.768	14	4.6%	22	5.9%	32	7.7%	0.086
Acute kidney injury	11	6.2%	20	11.8%	18	9.1%	0.348	29	3.8%	62	7.4%	32	8.6%	<0.001	9	3.0%	35	9.4%	39	9.4%	0.002
Electrolyte disturbance	5	2.8%	12	7.1%	14	7.1%	0.08	19	2.5%	45	5.4%	27	7.2%	<0.001	10	3.3%	30	8.0%	25	6.0%	0.185
Constipation or adynamic ileus	6	3.4%	8	4.7%	20	10.2%	0.006	28	3.7%	54	6.4%	26	7.0%	0.01	7	2.3%	24	6.4%	34	8.2%	0.001
Anemia	22	12.4%	27	15.9%	40	20.3%	0.04	164	21.7%	278	33.1%	115	30.7%	<0.001	59	19.4%	103	27.5%	110	26.4%	0.044
Transfusion	15	8.5%	26	15.3%	15	7.6%	0.726	189	25.0%	266	31.7%	62	16.6%	<0.001	61	20.1%	110	29.4%	69	16.6%	0.133
Urinary catheterization	4	2.3%	8	4.7%	5	2.5%	0.911	34	4.5%	37	4.4%	10	2.7%	0.199	8	2.6%	16	4.3%	14	3.4%	0.665
Malnutrition	5	2.8%	7	4.1%	25	12.7%	<0.001	20	2.6%	64	7.6%	41	11.0%	<0.001	10	3.3%	35	9.4%	53	12.7%	<0.001
Delirium	7	4.0%	9	5.3%	14	7.1%	0.182	21	2.8%	62	7.4%	30	8.0%	<0.001	16	5.3%	23	6.1%	38	9.1%	0.038
Pressure ulcers	9	5.1%	11	6.5%	8	4.1%	0.635	23	3.0%	34	4.0%	16	4.3%	0.244	10	3.3%	23	6.1%	20	4.8%	0.424

Pharmacologic or procedure-related complications	6	3.4%	10	5.9%	15	7.6%	0.08	69	9.1%	72	8.6%	38	10.2%	0.691	37	12.2%	40	10.7%	43	10.3%	0.450
In-hospital mortality	58	32.8%	60	35.3%	55	27.9%	0.299	104	13.8%	93	11.1%	41	11.0%	0.112	44	14.5%	38	10.2%	55	13.2%	0.731
Hospital length of stay (days)	7.1±6.1	6	6.7±6.7	4	9.4±16.1	5	0.58	12.9±8.4	11	10.5±10.1	9	9.6±5.9	8	<0.001	13±7	12	11.3±6.8	10	10.3±7	9	<0.001

Note: Continuous variables are expressed as mean ± standard deviation (median) and categorical variables as number (percentage). * Percentage of total number of surgeries (71.2% of patients).

Supplementary Table 5. Trends in the characteristics of hip fracture patients with a surgical delay >=48 hours or dead during hospital stay. Hip fracture admissions among centenarians in Spain, 2004-2020.

	Surgical delay >=48 hours							In-hospital death						
	2004-2010		2011-2015		2016-2020		p	2004-2010		2011-2015		2016-2020		p
Age (years)	101.2±1.6	101	101.2±1.5	101	101.2±1.6	101	0.902	101.4±1.7	101	101.5±1.7	101	101.5±1.8	101	0.496
Sex. female	360	79.3%	335	84.2%	416	83.4%	0.107	161	77.8%	161	81.3%	195	83.3%	0.141
Injury characteristics							0.022							0.690
Subtrochanteric fracture	36	7.9%	31	7.8%	32	6.4%		12	5.8%	13	6.6%	22	9.4%	
Pertrochanteric fracture	251	55.3%	205	51.5%	246	49.3%		115	55.6%	111	56.1%	108	46.2%	
Intracapsular fracture	167	36.8%	162	40.7%	221	44.3%		80	38.6%	74	37.4%	104	44.4%	
Number of chronic diseases	1.5±1.4	1	2.1±1.7	2	2.4±1.8	2	<0.001	1.7±1.2	2	2.4±1.6	2	2.4±1.9	2	<0.001
0	131	28.9%	75	18.8%	73	14.6%		37	17.9%	22	11.1%	38	16.2%	
1	131	28.9%	91	22.9%	106	21.2%		61	29.5%	41	20.7%	53	22.6%	
2	94	20.7%	96	24.1%	108	21.6%		56	27.1%	47	23.7%	42	17.9%	
3	66	14.5%	67	16.8%	78	15.6%		37	17.9%	52	26.3%	36	15.4%	
>3	32	7.0%	69	17.3%	134	26.9%		16	7.7%	36	18.2%	65	27.8%	
Multimorbidity	192	42.3%	232	58.3%	320	64.1%	<0.001	109	52.7%	135	68.2%	143	61.1%	0.085
Charlson Comorbidity index (CCI)	0.6±1	0	0.9±1.2	0	1.2±1.4	1	<0.001	0.9±1.1	1	1.3±1.4	1	1.4±1.6	1	<0.001
Severe comorbidity (CCI ≥ 3)	29	6.4%	42	10.6%	85	17.0%	<0.001	23	11.1%	39	19.7%	57	24.4%	<0.001
Surgical delay (days)*	5.3±2.5	5	4.9±2.4	4	4.8±2.5	4	<0.001	3.9±3.6	3	3±3.2	2	2.5±2.4	2	0.001
<24 horas								14	0.1272727273	8	0.09090909091	27	0.1525423729	

[24-48) horas								15	0.1363636364	22	0.25	44	0.2485875706	
>=48 horas								81	0.7363636364	58	0.6590909091	106	0.5988700565	
In-hospital complications														
Respiratory tract infection	17	3.7%	17	4.3%	27	5.4%	0.214	22	10.6%	22	11.1%	35	15.0%	0.162
Urinary tract infection	13	2.9%	25	6.3%	42	8.4%	<0.001	7	3.4%	12	6.1%	14	6.0%	0.228
Acute respiratory failure	22	4.8%	25	6.3%	41	8.2%	0.035	41	19.8%	29	14.6%	40	17.1%	0.476
Acute kidney injury	16	3.5%	36	9.0%	43	8.6%	0.003	25	12.1%	41	20.7%	44	18.8%	0.070
Electrolyte disturbance	19	4.2%	28	7.0%	35	7.0%	0.072	12	5.8%	23	11.6%	30	12.8%	0.016
Constipation or adynamic ileus	20	4.4%	29	7.3%	28	5.6%	0.447	10	4.8%	16	8.1%	23	9.8%	0.050
Anemia	94	20.7%	118	29.6%	171	34.3%	<0.001	50	24.2%	56	28.3%	75	32.1%	0.067
Transfusion	109	24.0%	124	31.2%	123	24.6%	0.875	57	27.5%	55	27.8%	58	24.8%	0.505
Urinary catheterization	23	5.1%	24	6.0%	22	4.4%	0.627	6	2.9%	9	4.5%	16	6.8%	0.054
Malnutrition	19	4.2%	40	10.1%	69	13.8%	<0.001	6	2.9%	7	3.5%	19	8.1%	0.011
Delirium	16	3.5%	31	7.8%	43	8.6%	0.002	13	6.3%	11	5.6%	27	11.5%	0.037
Pressure ulcers	20	4.4%	23	5.8%	19	3.8%	0.635	6	2.9%	7	3.5%	8	3.4%	0.766
Pharmacologic or procedure-related complications	53	11.7%	47	11.8%	54	10.8%	0.674	34	16.4%	34	17.2%	46	19.7%	0.371
In-hospital mortality	60	13.2%	38	9.5%	71	14.2%	0.599							
Hospital length of stay (days)	15.6±9.3	13	13.8±7.5	12	11.8±6.6	10	<0.001	10.3±8.6	8	8.9±7.2	7	9.3±9.7	7	0.058

Note: Continuous variables are expressed as mean ± standard deviation (median) and categorical variables as number (percentage). * Percentage of total number of surgeries (71.2% of patients).
